# Metformin-Associated Lactic Acidosis in Individuals Without Chronic Kidney Disease on Therapeutic Dose: A Case Report

**DOI:** 10.7759/cureus.48683

**Published:** 2023-11-12

**Authors:** Masafumi Fukuda, Nobuhisa Hirayu, Masakazu Nabeta, Masafumi Goto, Osamu Takasu

**Affiliations:** 1 Intensive Care Unit, Advanced Emergency and Critical Care Center, Kurume University Hospital, Kurume, JPN

**Keywords:** chronic kidney disease, recommended daily dose, elderly, acute kidney injury, metformin-associated lactic acidosis

## Abstract

Metformin-associated lactic acidosis (MALA) is a severe side effect of metformin treatment. We encountered an exceedingly rare case of MALA in a patient taking metformin at recommended doses who had no risk factors except for advanced age. A 77-year-old male with a diagnosis of lactic acidosis was referred to our facility. He was taking 250 mg/day of metformin for diabetes. Although he had no pre-existing chronic kidney disease, he developed acute kidney injury upon admission, leading to the diagnosis of MALA based on the test results and history of metformin use. His lactic acidosis improved without extracorporeal treatment through metformin discontinuation and proper circulatory management. When encountering patients with unexplained lactic acidosis, it is important to consider MALA as part of the differential diagnosis and to confirm the patient's medication history. Specifically, when metformin use is identified, attention should be directed toward the potential for MALA.

## Introduction

In a patient population without a specific diagnosis, hyperlactatemia is considered to be associated with a poor prognosis [1]. Although elevated lactate levels can occur in various diseases and conditions, in cases of hyperlactatemia without concomitant circulatory failure, thiamine deficiency, presence of toxins, and medication use should be considered [2]. Metformin-associated lactic acidosis (MALA) is an exceedingly rare condition with a serious side effect affecting patients receiving metformin [3]. While the estimated glomerular filtration rate (eGFR) <30 mL/min/1.73 m^2^ in chronic kidney disease [4] and medication overuse are considered risk factors for metformin-associated lactic acidosis (MALA), in the present case, we experienced an exceedingly rare occurrence of MALA despite the use of metformin at recommended doses and normal kidney function [5]. The patient provided written consent for the presentation of their case in the present study.

## Case presentation

A 77-year-old male visited his primary care physician due to nausea and fatigue along with decreased oral intake due to loss of appetite. He had a history of partial gastrectomy for gastric cancer and had been taking the same oral medications, including 20 mg olmesartan, 5 mg amlodipine, 25 mg alogliptin, 250 mg metformin, and 20 mg febuxostat, for at least the past six months to manage hypertension, diabetes, and hyperuricemia. There had been no changes or adjustments in his medication regimen during this period. Two months ago, the renal function was eGFR 77.5 mL/min/1.73 m², and six months ago, it was eGFR 74.2 mL/min/1.73 m², with no history of obvious chronic kidney disease (CKD). Additionally, there is no history of hepatitis infection, and there were no abnormalities in liver function on blood tests. During the visit to his primary care physician, he remained alert, had no decrease in blood pressure, and had a normal blood glucose level of 111 mg/dL. The arterial blood gas showed a pH of 7.295 (normal range: 7.35 to 7.45), bicarbonate level of 13.5 mmol/L (normal range: 22 to 28 mmol/L), base excess of -11.7 mmol/L (normal range: -2.0 to 2.0 mmol/L), and a lactate level of 5.76 mmol/L (normal range: 0.56 to 1.39 mmol/L), leading to the diagnosis of lactic acidosis. Furthermore, due to the presence of hyperkalemia, treatment with calcium gluconate, glucose, and insulin (glucose-insulin therapy) was initiated, along with the intravenous administration of Ringer's solution, and the patient was transferred to our facility. At the time of admission to our facility, the patient’s blood pressure was 146/58 mmHg and his pulse rate was 100/min. He was alert with a body temperature of 37.4°C. No pallor, coldness, or moistness of the fingertips was observed, and there were no clinical signs suggesting circulatory insufficiency. Table 1 shows the blood test results at the time of admission to our facility.

**Table 1:** Laboratory data on admission. WBC: white blood cell; RBC: red blood cell; Hb: hemoglobin; Ht: hematocrit; Plt: platelet; pH: power of hydrogen; PaCO_2_: partial pressure of arterial carbon dioxide; PaO_2_: partial pressure of arterial oxygen; HCO_3_: hydrogen carbonate; BE: base excess; Na: sodium; K: potassium; Cl: chloride; AST: aspartate aminotransferase; ALT: alanine aminotransferase; TP: total protein; T.bil: total bilirubin; BUN: blood urea nitrogen; Cre: creatinine; CRP: c-reactive protein; HbA1c: glycated hemoglobin

Biochemical analysis			Reference range
Blood cell count	WBC	21,500/μL	3,300 to 8,600/µL
	RBC	3.31×10^6^/μL	4.35 to 4.92×10^6^/µL
	Hb	8.6 g/dL	11.6 to 14.8 g/dL
	Ht	28.60%	35.1% to 44.4%
	Plt	289×10^3^/μL	158 to 348×10^3^/µL
Blood gas analysis	pH	7.395	7.380 to 7.460
	PaCO_2_	29.3 mmHg	32.0 to 46.0 mmHg
	PaO_2_	79.0 mmHg	74.0 to 109.0 mmHg
	HCO_3−_	17.9 mmol/L	21.0 to 29.0 mmol/L
	BE	-6.1 mmol/L	-2.0 to 2.0 mmol/L
	Lactic acid	4.9 mmol/L	0.56 to 1.39 mmol/L
Biochemistry	Na	132 mEq/L	138 to 145 mEq/L
	K	5.6 mEq/L	3.6 to 4.8 mEq/L
	Cl	95 mEq/L	101 to 108 mEq/L
	AST	61 IU/L	13 to 30 IU/L
	ALT	17 IU/L	10 to 30 IU/L
	TP	5.2 g/dL	6.6 to 8.1 g/dL
	T.Bil	0.2 mg/dL	0.4 to 1.2 mg/dL
	BUN	40 mg/dL	8 to 20 mg/dL
	Cre	2.52 mg/dL	0.65 to 1.07 mg/dL
	CRP	1.14 mg/dL	≤0.14 mg/dL
	HbA1c	6.10%	4.9 to 6.0 mg/dL
	Glucose	222 mg/dL	73 to 109 mg/dL
	Thiamine	43 ng/mL	24 to 66 ng/mL
	3-hydroxybutyric acid	2644 µmol/L	<74 µmol/L

**Figure 1:**
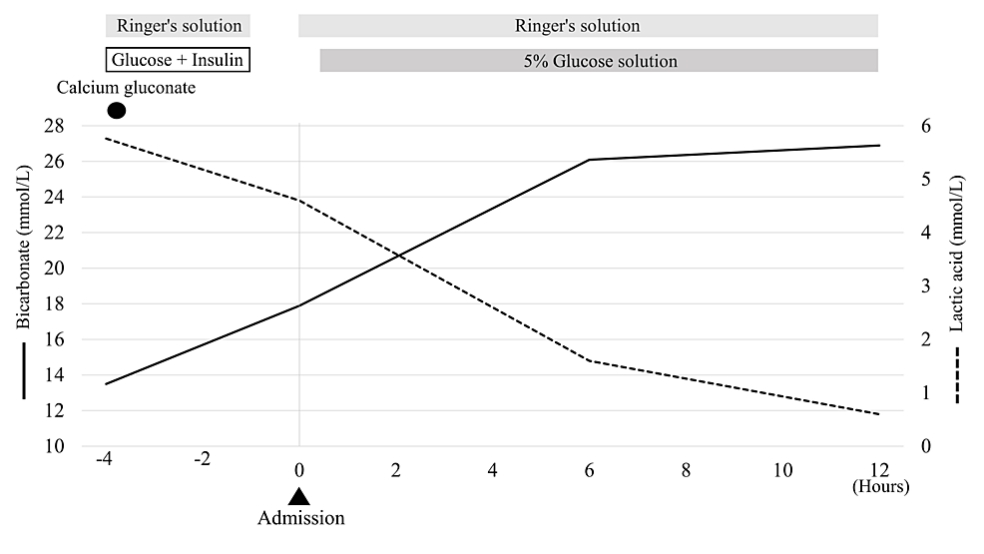
Effect of treatment on the plasma lactic acid and bicarbonate levels. The image shows the trend in the plasma lactic acid and bicarbonate levels after treatment. At 12 hours after starting treatment, both the bicarbonate and lactic acid levels improved. The bicarbonate level improved to 26.9 mmol/L, and the lactic acid level decreased to 0.6 mmol/L.

The blood urea nitrogen and creatinine levels were 40 mg/dL (normal range: 8-20 mg/dL) and 2.52 mg/dL (normal range: 0.65-1.07 mg/dL), respectively, both indicating elevated levels. Due to the significantly elevated white blood cell count of 21,500/µL (normal range: 3,300 to 8,600 /µL), we suspected an infection and conducted urine culture and chest and abdominal computed tomography scans, but no clear abnormalities were observed. Given the history of reduced food intake due to decreased appetite and elevated blood urea nitrogen and creatinine levels, dehydration was suspected. The thiamine levels were within the normal range, suggesting the presence of lactic acidosis. Based on these test results, his history of metformin use, and a Naranjo Adverse Drug Reaction probability scale score of five points, he was diagnosed with metformin-associated lactic acidosis (MALA), leading to his admission to the intensive care unit (ICU) for treatment [6]. The treatment progress is shown in Figure 1.

Owing to the increased blood ketone levels, the patient was considered to have had ketosis as a complication. Therefore, while administering the Ringer's solution, a glucose solution (5%) was additionally added to treat ketosis after admission. As a result, his lactic acidosis improved smoothly, and the arterial blood gas values normalized. Consequently, the patient was discharged from the ICU within 12 hours of starting treatment. Given that his creatinine level also improved to 0.74 mg/dL on the fifth day of treatment, the patient was discharged home on the same day. A reevaluation was conducted at two weeks after discharge with metformin discontinuation, but no recurrence of lactic acidosis or worsening of renal function was observed.

## Discussion

Metformin is the first-line treatment of choice for type 2 diabetes due to its high efficacy in lowering glycated hemoglobin levels and reducing the risk of cardiovascular morbidity and mortality [7]. While the incidence of lactic acidosis in metformin users is extremely rare at nine per 100,000 person-years [8], it is important to note that the mortality rate for MALA is high, ranging from 30% to 50% [9]. Hence, it is a significant complication to be aware of when using metformin. Nonetheless, due to its notably low frequency and in the absence of a history of severe CKD or excessive metformin intake, differentiating MALA, even when lactic acidosis is present, is not straightforward. Symptoms of MALA include drowsiness, nausea, vomiting, abdominal pain, diarrhea, and in more severe cases, low blood pressure and low body temperature [10]. It is important to note that there may not be specific symptoms. Despite the challenges in differentiation, the importance of diagnosing MALA remains substantial. While it may be difficult to differentiate the disease, the early diagnosis of MALA, even in the presence of lactic acidosis, could potentially reduce the mortality rate in patients with severe MALA [11]. In fact, when diagnosing MALA, measuring the blood concentration of metformin can be valuable; thus, it is important to measure the metformin concentration when MALA is suspected in a patient [12]. However, measuring metformin concentration is not easy and cannot be quickly done in any facility. In other words, when unexplained lactic acidosis is observed, it is necessary to confirm the history of metformin use. The commonly known risk factors for MALA include poor renal function, impaired hepatic metabolism, shock, alcohol use, hypoxic state, sepsis, and advanced age [13]. While the present case lacked the typical risk factors of MALA, apart from the advanced age, the results of the admission tests revealed acute kidney injury (AKI). There have been reports suggesting an increased risk of lactic acidosis with severe AKI in metformin users [14]. Therefore, the presence of AKI, in addition to pre-existing CKD, is considered a crucial factor in MALA diagnosis. Since metformin is excreted mostly unchanged by the kidneys, a decrease in the glomerular filtration rate leads to reduced metformin clearance [15]. In other words, regardless of whether there is pre-existing CKD or AKI, attention should be paid to renal function when diagnosing lactic acidosis. Due to metformin's low molecular weight of 165 and minimal protein binding, along with its ability to easily pass through dialysis membranes [16], extracorporeal treatment such as hemodialysis should be considered as a therapeutic option for MALA cases [17]. However, in the present case, the discontinuation of metformin and proper circulatory management led to a swift recovery without the need for extracorporeal treatment, such as hemodialysis. Early detection and diagnosis of MALA is particularly crucial, as this may allow for treatment similar to the present case, avoiding costly advanced medical procedures, such as extracorporeal treatment. In the recent guidelines, metformin is no longer contraindicated for patients with eGFR<45 mL/min [18]. Therefore, as there is still a possibility that a certain number of patients may develop MALA in the future, MALA should be considered a differential diagnosis for patients using metformin who developed lactic acidosis.

## Conclusions

In patients without chronic kidney disease who are receiving metformin, it is important to be aware of the possibility of them developing MALA. When encountering lactic acidosis of unknown etiology in patients with diabetes, it is necessary to verify the medications and be mindful of the possibility of MALA.
